# Supplementary figures and images for: Microbial Community Composition and Diversity via 16S rRNA Gene Amplicons: Evaluating the Illumina Platform
Source: PLoS One. 2015 Feb 3;10(2):e0116955. doi: 10.1371/journal.pone.0116955 (PMC4315398; doi:10.1371/journal.pone.0116955)

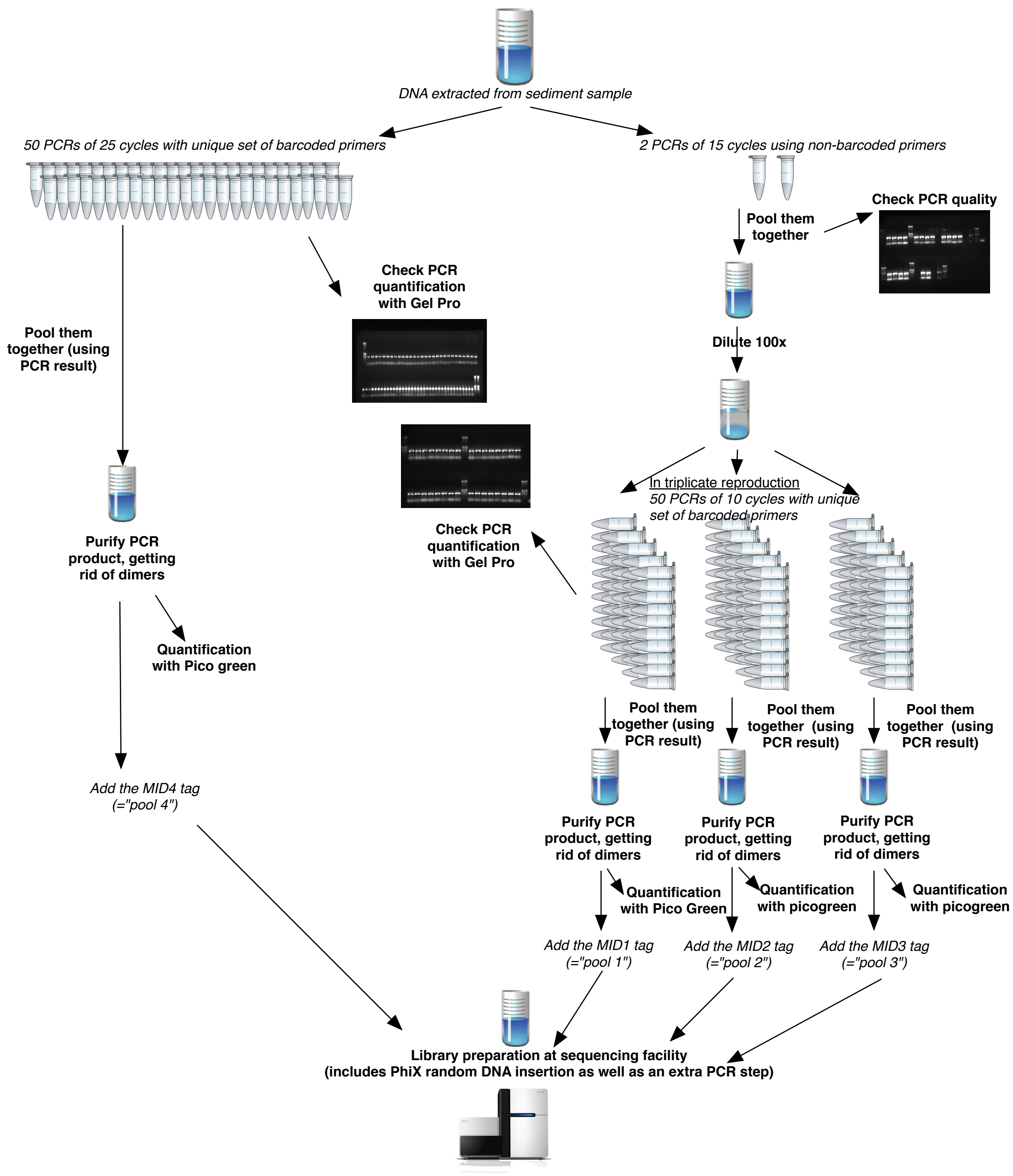

Supplement: S1 Fig — This figure details the outline of the experimental design. The extra fifth pool where the updated protocol was used is not represented. Other samples run with pico green individual quantification are not shown either. The 454 sample is not shown. (PDF) [file pone.0116955.s001.pdf]

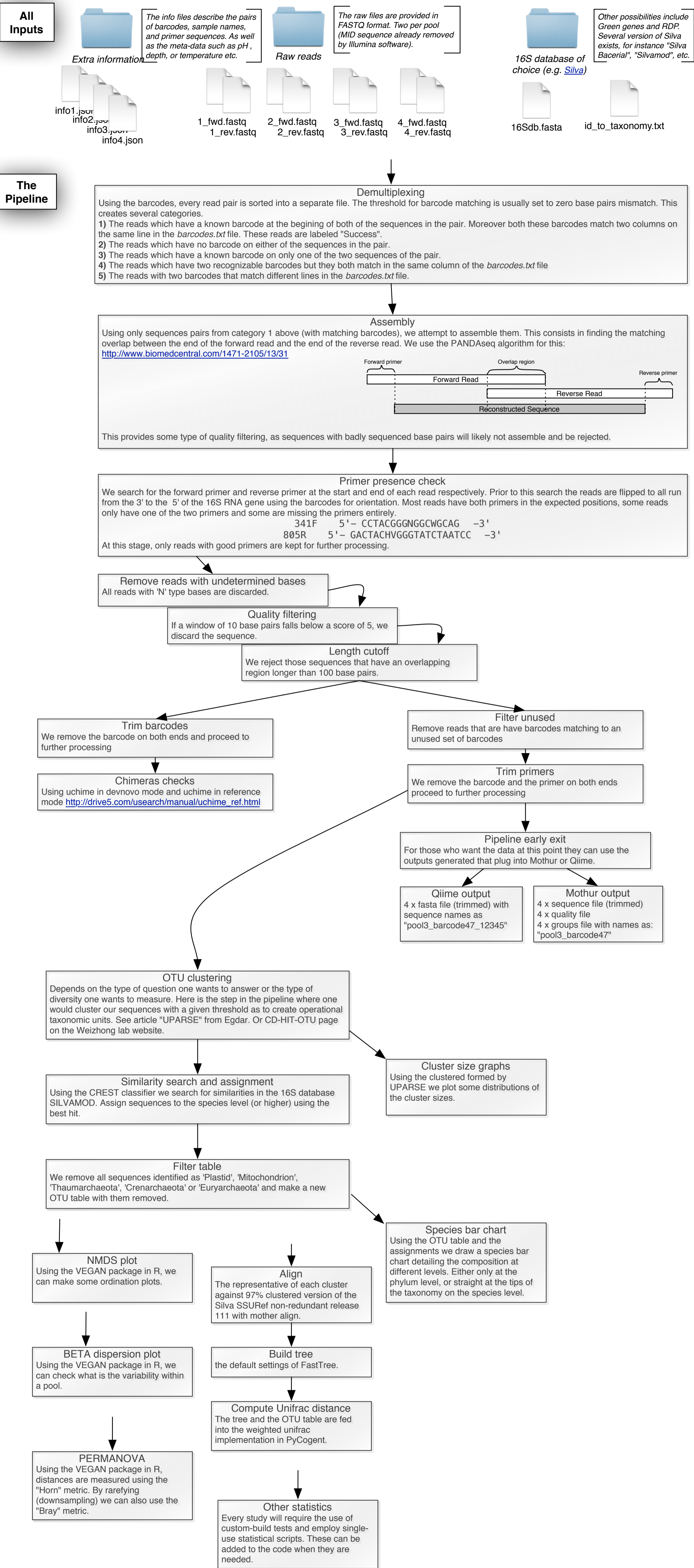

Supplement: S2 Fig — This figure summarizes in flow chart the bioinformatics pipeline that was applied to the data. The code was written in python and is available at http://github.com/limno/illumitag/ under an MIT license. (PDF) [file pone.0116955.s002.pdf]

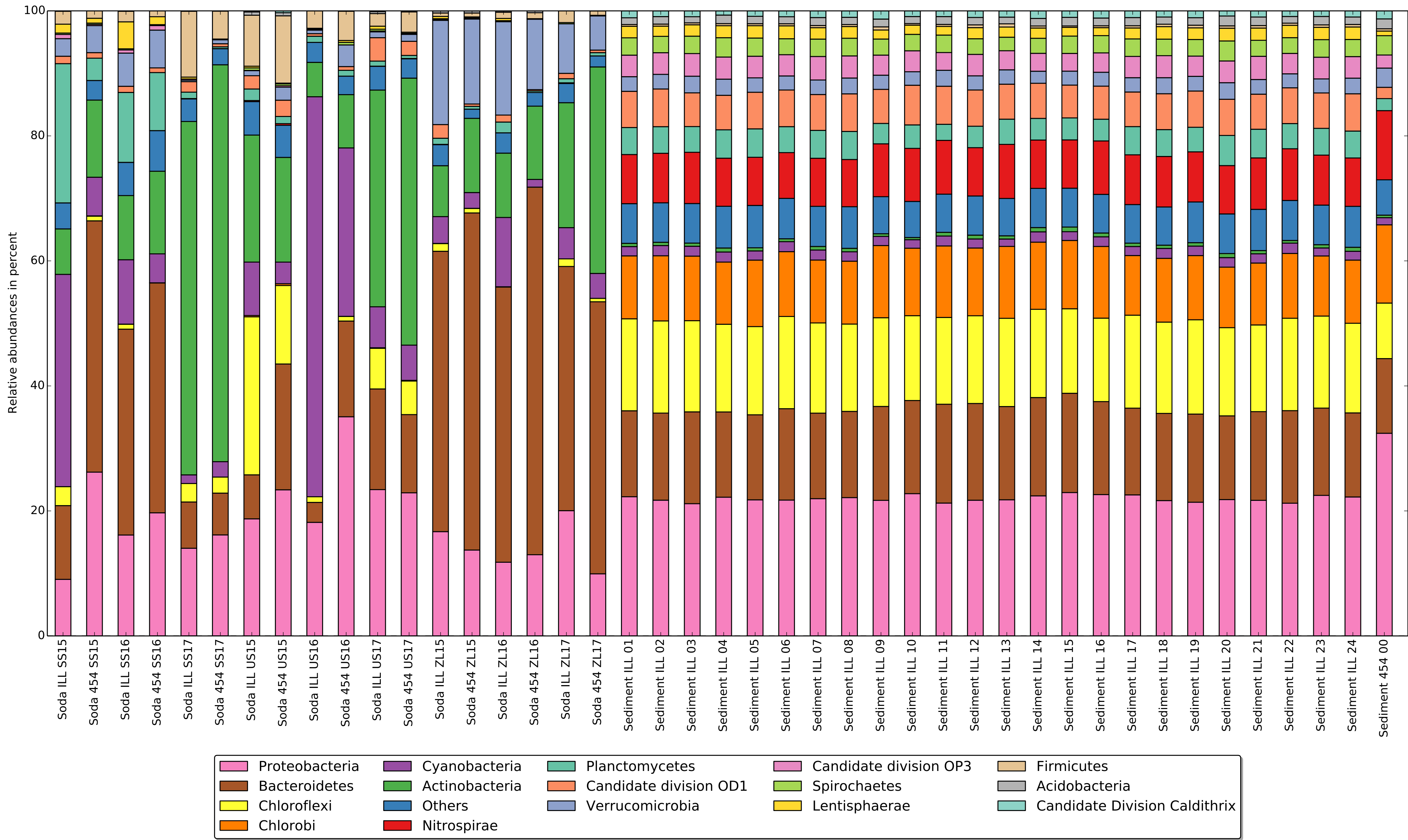

Supplement: S7 Fig — Are represented here 24 replicates of our test sediment sample sequenced on the Illumina platform (“Sediment ILL”) as well as the same sample sequenced on a 454 machine (“Sediment 454”). In addition, 9 soda lakes samples were independently sequenced on Illumina and 454. The species relative abundances per sample classified at the phylum level is shown in a barstack. (PDF) [file pone.0116955.s007.pdf]

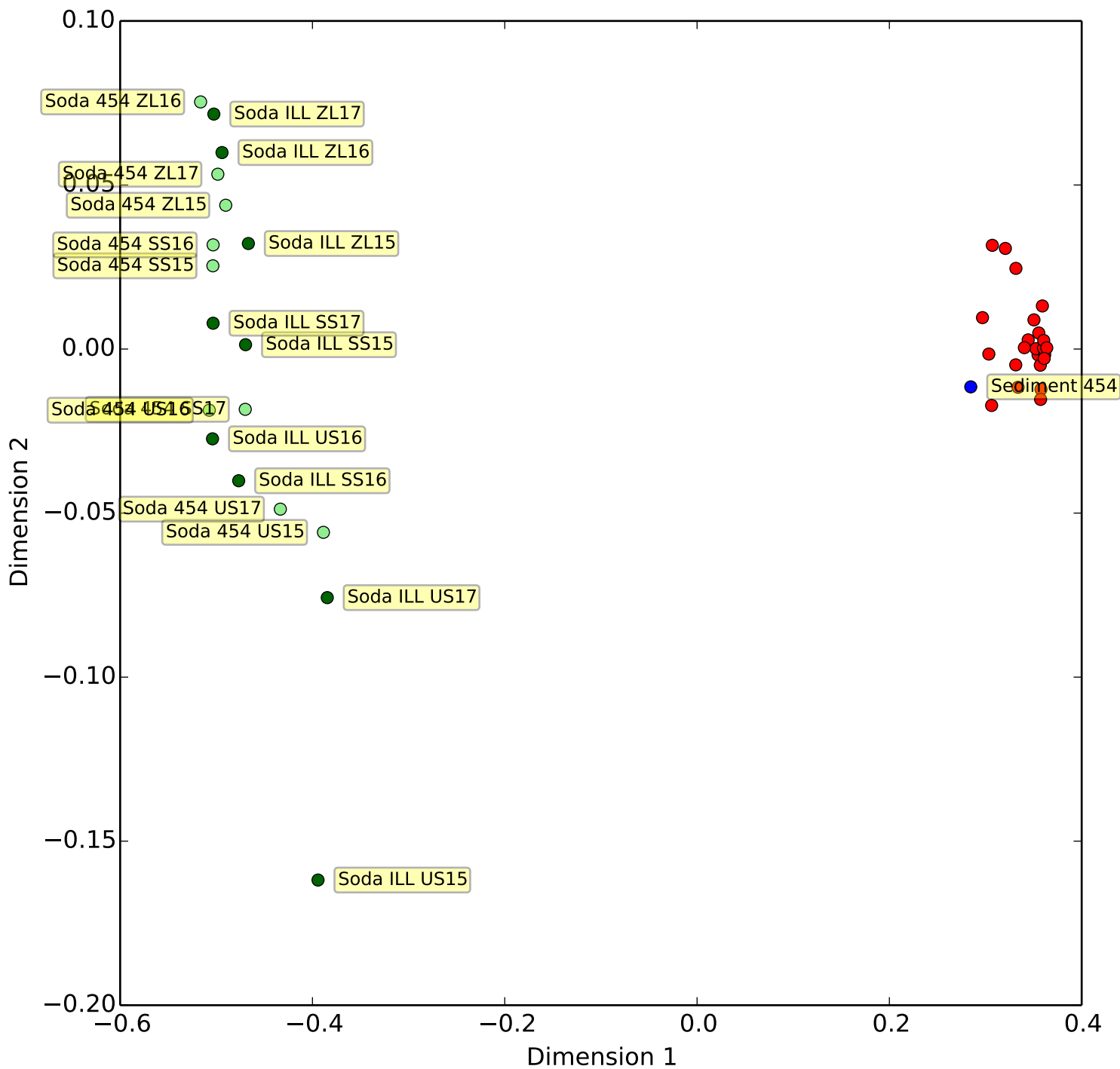

Supplement: S8 Fig — Are represented here 24 replicates of our test sediment sample sequenced on the Illumina platform (red points without labels) as well as the same sample sequenced on a 454 machine (blue point labeled “Sediment 454”). In addition, 9 soda lakes were independently sequenced on Illumina and 454 (green labels “Soda 454” and “Soda ILL”). The Unifrac metric is used to compute the distance matrix from which the NMDS is calculated. (PDF) [file pone.0116955.s008.pdf]
