# Supplementary material for: Microbial Community Composition and Diversity via 16S rRNA Gene Amplicons: Evaluating the Illumina Platform
Source: PLoS One. 2015 Feb 3;10(2):e0116955. doi: 10.1371/journal.pone.0116955 (PMC4315398; doi:10.1371/journal.pone.0116955)
Supplement: S3 Fig — Quality distribution plots for each of the five Illumina pools of sediment data as generated by FastQC and based on the raw FASTQ files. Forward and reverse reads are shown seperately. (PDF) [file pone.0116955.s003.pdf]

Quality distribution

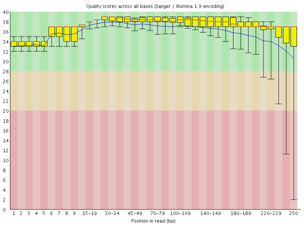

(a) Pool 1 forward reads

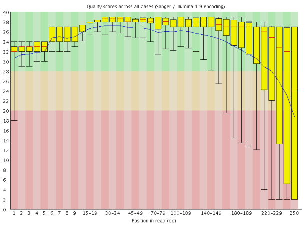

(b) Pool 1 reverse reads

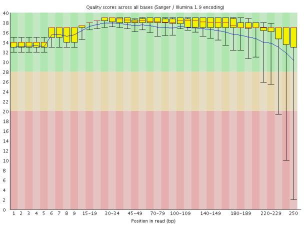

(c) Pool 2 forward reads

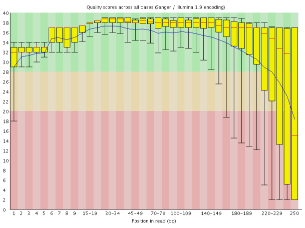

(d) Pool 2 reverse reads

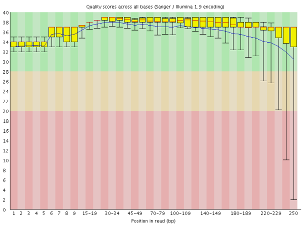

(e) Pool 3 forward reads

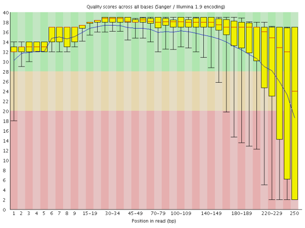

(f) Pool 3 reverse reads

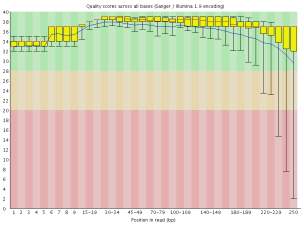

(g) Pool 4 forward reads

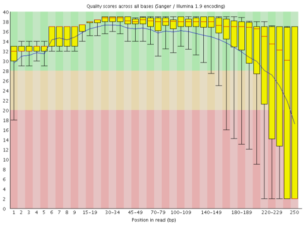

(h) Pool 4 reverse reads

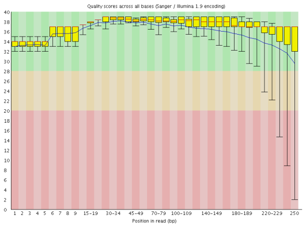

(i) Pool 5 forward reads

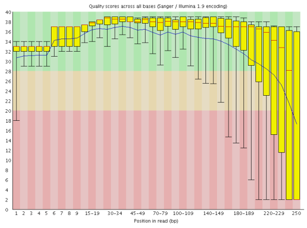

(j) Pool 5 reverse reads
