# Supplementary material for: Microbial Community Composition and Diversity via 16S rRNA Gene Amplicons: Evaluating the Illumina Platform
Source: PLoS One. 2015 Feb 3;10(2):e0116955. doi: 10.1371/journal.pone.0116955 (PMC4315398; doi:10.1371/journal.pone.0116955)
Supplement: S4 Fig — Distribution of sequences that have matching barcodes for every barcode identifier in each of the five Illumina pools of sediment data. (PDF) [file pone.0116955.s004.pdf]

Barcode distribution

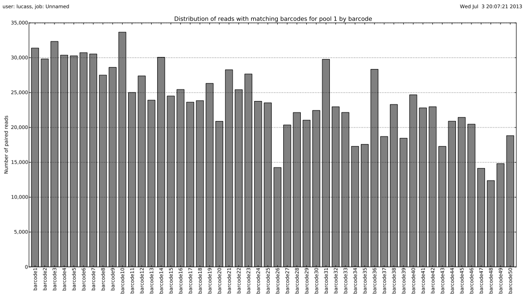

(a) Pool 1 (Two-step PCR I)

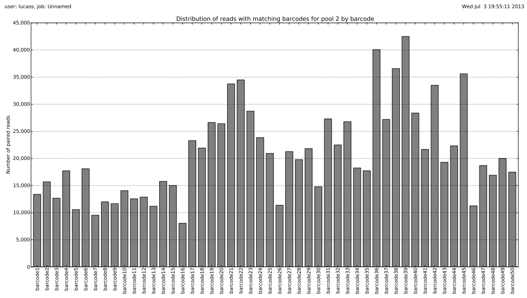

(b) Pool 2 (Two-step PCR II)

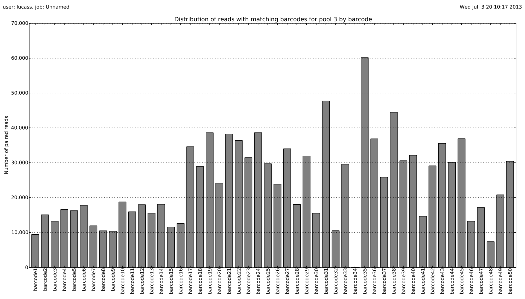

(c) Pool 3 (Two-step PCR III)

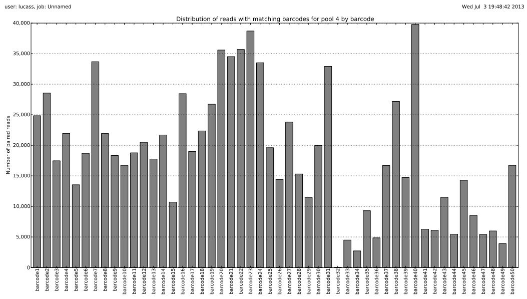

(d) Pool 4 (Single-step PCR)

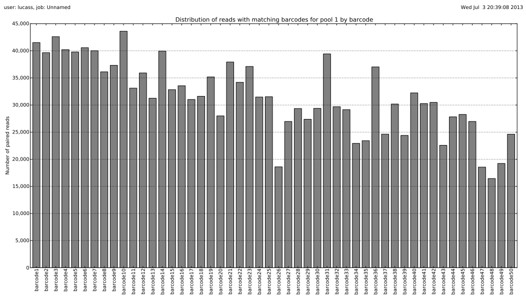

(e) Pool 5 (Updated Chemistry)
