# Supplementary material for: Microbial Community Composition and Diversity via 16S rRNA Gene Amplicons: Evaluating the Illumina Platform
Source: PLoS One. 2015 Feb 3;10(2):e0116955. doi: 10.1371/journal.pone.0116955 (PMC4315398; doi:10.1371/journal.pone.0116955)
Supplement: S5 Fig — This figure shows the relative proportions of mismatched barcodes pairs for every pool. For every one of 100 barcodes, we compute a comparision with every one of the 100 barcodes exlucding the two cases where the two barcodes are correctly matching (e.g. barcode2F-barcode2R) and where the two barcodes are the same (e.g. barcode16R-barcode16R). This amounts to a total of 9800 points on the graph. One has to understand that considering the pair “barcode1R-barcode15F” will not produce the same point as the pair “barcode15F-barcode1R”. Hence, for every disctinct sequence of two mismatching barcodes (e.g. barcode1R-barcode15F) the position on the X axis is calculated by dividing the number of correct matches containing the first barcode (e.g. barcode1R) by the number of all correct matches. The position on the Y axis is computed by dividing the number of reads containing these two particular barcodes in this specific order by the number of mismatches involving the second barcode (e.g. barcode15F). Every point deviating significativly from the regression has its label drawn and is potentially suspicous. (PDF) [file pone.0116955.s005.pdf]

Match and mismatch regression

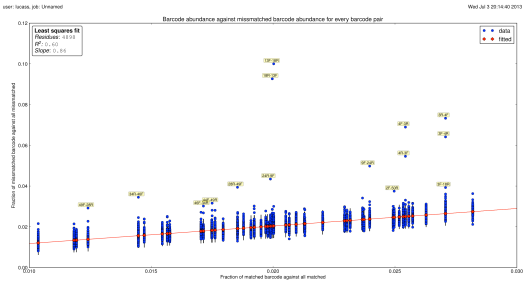

(a) Pool 1 (Two-step PCR I)

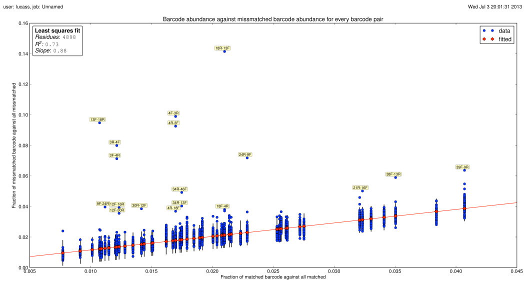

(b) Pool 2 (Two-step PCR II)

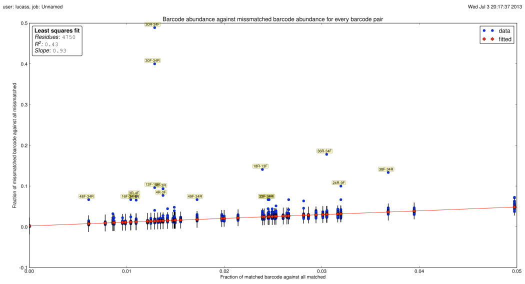

(c) Pool 3 (Two-step PCR III)

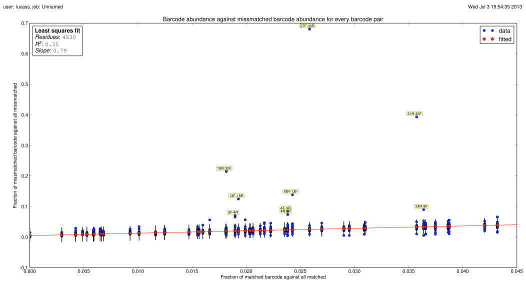

(d) Pool 4 (Single-step PCR)

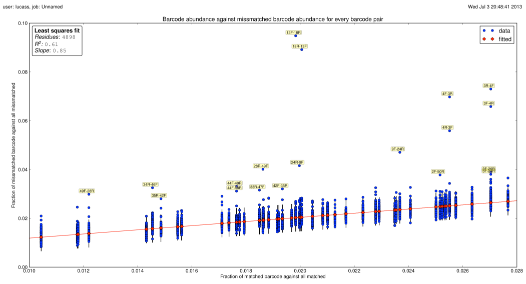

(e) Pool 5 (Updated Chemistry)
