# Supplementary material for: Microbial Community Composition and Diversity via 16S rRNA Gene Amplicons: Evaluating the Illumina Platform
Source: PLoS One. 2015 Feb 3;10(2):e0116955. doi: 10.1371/journal.pone.0116955 (PMC4315398; doi:10.1371/journal.pone.0116955)
Supplement: S6 Fig — Proportions of assembled and unassembled sequence pairs. Reads that marked as “Low quality” did assemble, but the assembly was not sufficiently good and below the threshold set at 0.6. Sequences that marked as “Unassembled” could not be assembled at all and no overlap could be determined. (PDF) [file pone.0116955.s006.pdf]

Assembled against unassembled

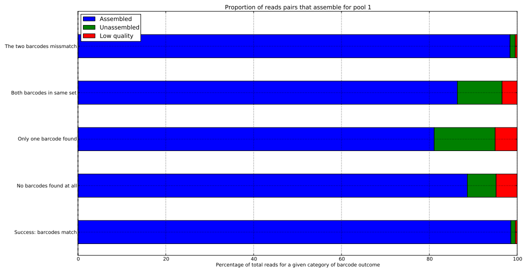

(a) Pool 1 (Two-step PCR I)

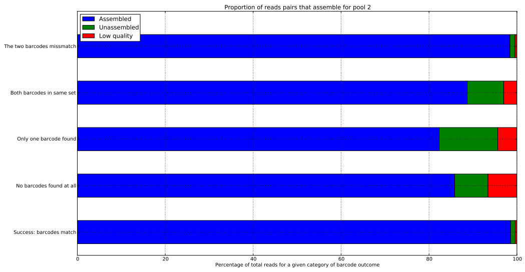

(b) Pool 2 (Two-step PCR II)

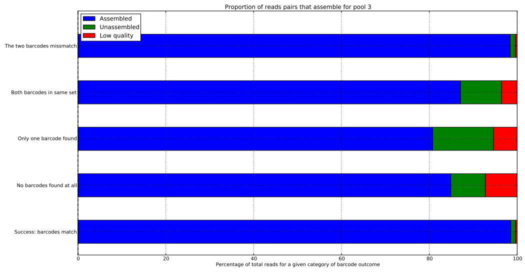

(c) Pool 3 (Two-step PCR III)

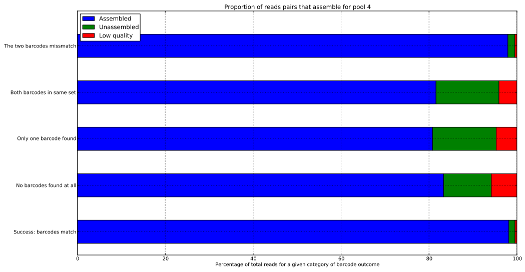

(d) Pool 4 (Single-step PCR)

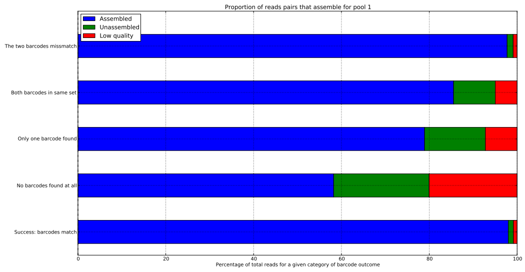

(e) Pool 5 (Updated Chemistry)
