# Supplementary material for: Microbial Community Composition and Diversity via 16S rRNA Gene Amplicons: Evaluating the Illumina Platform
Source: PLoS One. 2015 Feb 3;10(2):e0116955. doi: 10.1371/journal.pone.0116955 (PMC4315398; doi:10.1371/journal.pone.0116955)
Supplement: S9 Fig — OTU accumulation curves for every of the three OTU clustering methods. To draw these graphs, the OTUs appearing in at least X% of samples are considered and all reads pertaining to these OTUs are sumed up to one number. X is varied from 0% to 100% on the horizontal axis and the vertical axis represents the sum computed. (PDF) [file pone.0116955.s009.pdf]

OTU accumulation curves

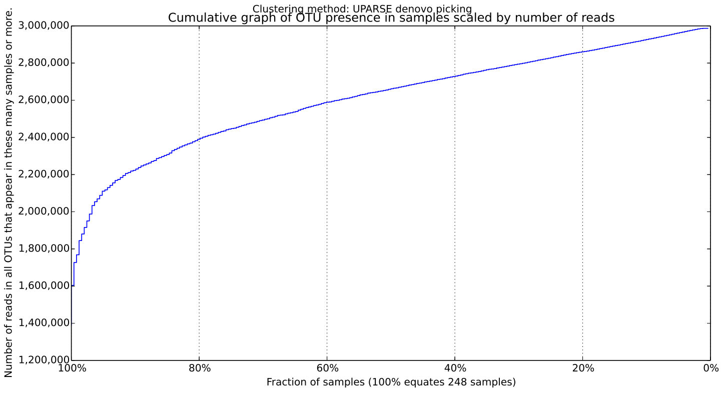

(a) UPARSE clustering method

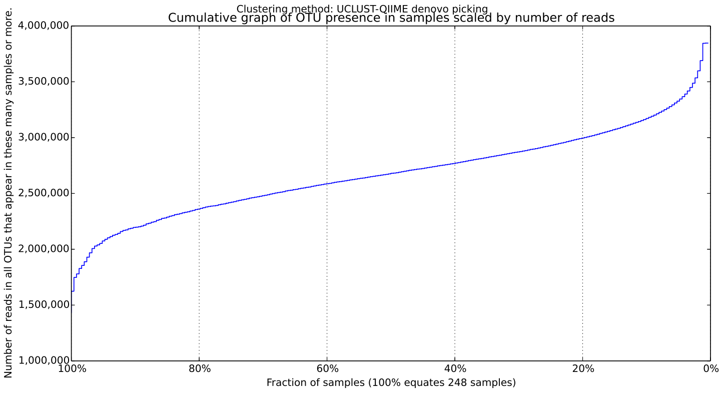

(b) UCLUST clustering method

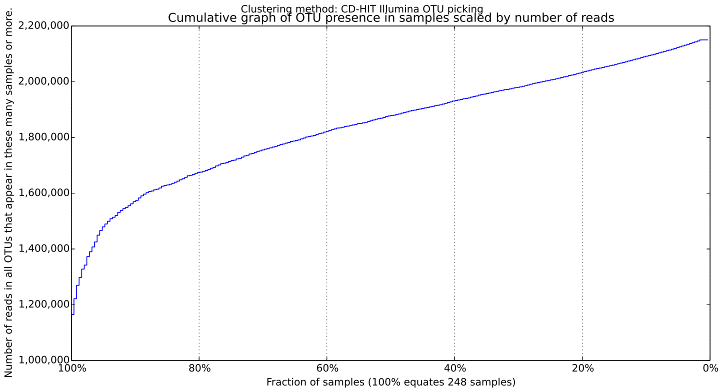

(c) CD-HIT-OTU clustering method
