# Supplementary material for: Microbial Community Composition and Diversity via 16S rRNA Gene Amplicons: Evaluating the Illumina Platform
Source: PLoS One. 2015 Feb 3;10(2):e0116955. doi: 10.1371/journal.pone.0116955 (PMC4315398; doi:10.1371/journal.pone.0116955)
Supplement: S10 Fig — A comparison is made between the diversity measures obtained for each sample that were both sequenced on Illumina and 454 platforms. Each line shows the fit of a linear models to the one of five different alpha-diversity estimates (A to E). Each dot represents one of the seven samples with more than 4391 reads (others not included). (PDF) [file pone.0116955.s010.pdf]

Regressions for comparing 454 and Illumina samples

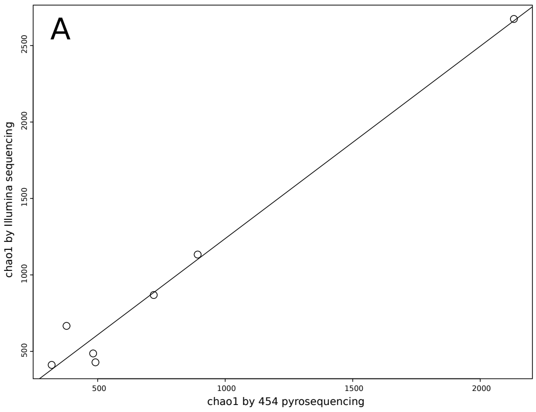

(a) Chao1

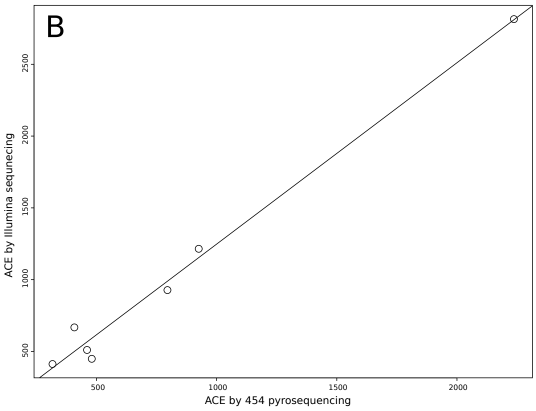

(b) ACE

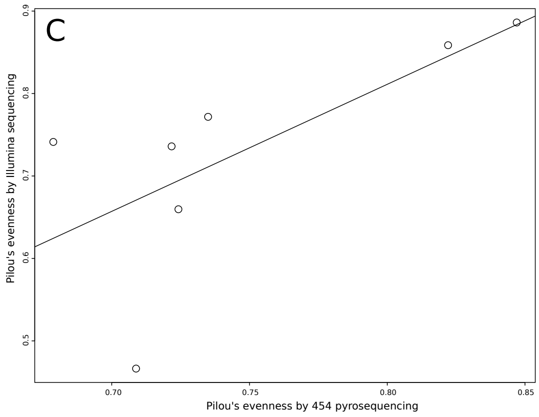

(c) Pilou's

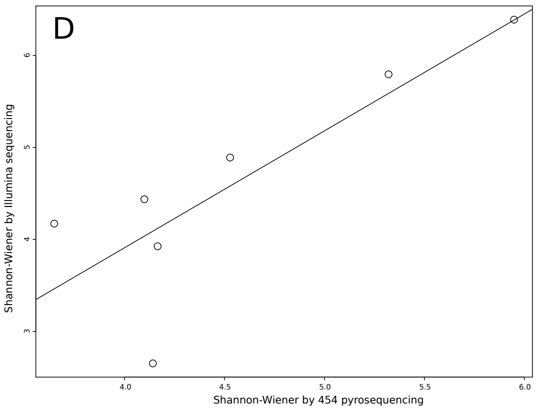

(d) Shannon Wiener

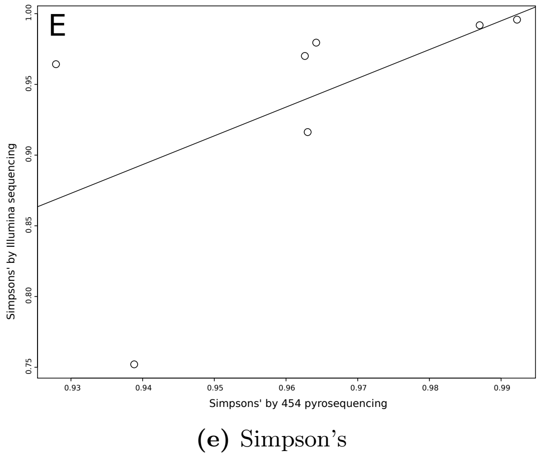

(e) Simpson's
