# Supplementary material for: Microbial Community Composition and Diversity via 16S rRNA Gene Amplicons: Evaluating the Illumina Platform
Source: PLoS One. 2015 Feb 3;10(2):e0116955. doi: 10.1371/journal.pone.0116955 (PMC4315398; doi:10.1371/journal.pone.0116955)
Supplement: S11 Fig — Statistics on the second illumina run, where the method is further used and put in production for various other projects (PDF) [file pone.0116955.s011.pdf]

# Production run 2

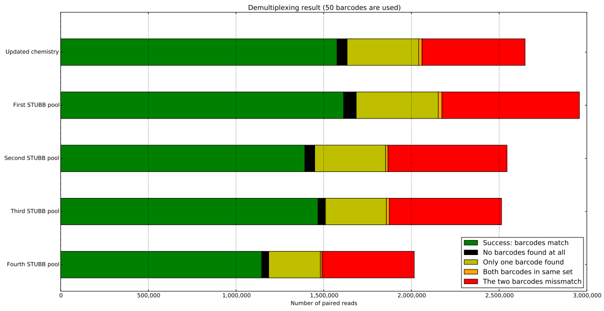


**(a)** Distribution of barcodes matching and mismatching

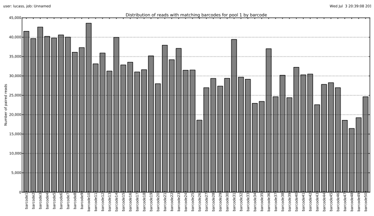


**(b)** Pool 1

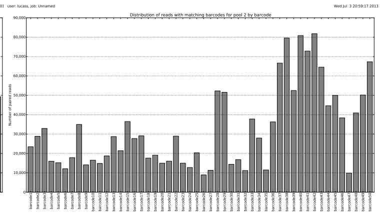


**(c)** Pool 2

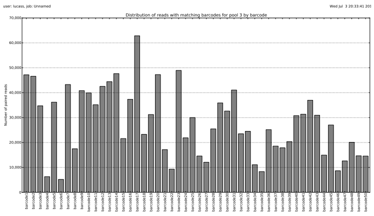


**(d)** Pool 3

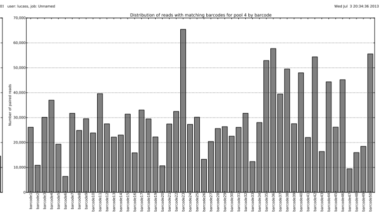


**(e)** Pool 4

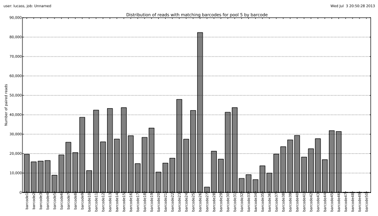


**(f)** Pool 5
