# Supplementary material for: Microbial Community Composition and Diversity via 16S rRNA Gene Amplicons: Evaluating the Illumina Platform
Source: PLoS One. 2015 Feb 3;10(2):e0116955. doi: 10.1371/journal.pone.0116955 (PMC4315398; doi:10.1371/journal.pone.0116955)
Supplement: S12 Fig — Statistics on the third illumina run, where the method is further used and put in production for various other projects. (PDF) [file pone.0116955.s012.pdf]

Production run 3

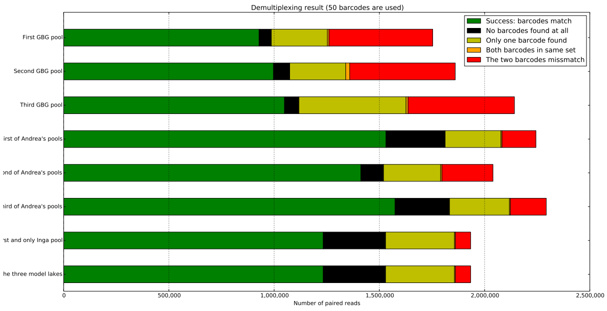

(a) Distribution of barcodes matching and mismatching

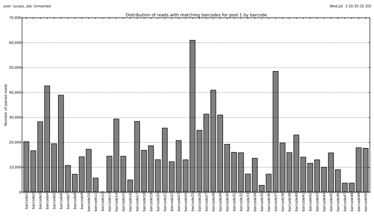

(b) Pool 1

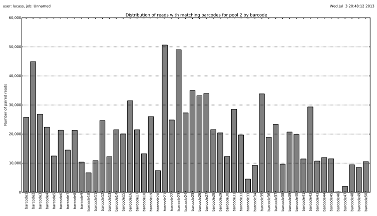

(c) Pool 2

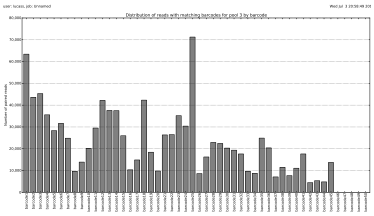

(d) Pool 3

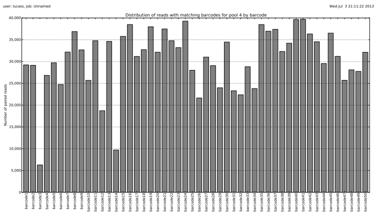

(e) Pool 4

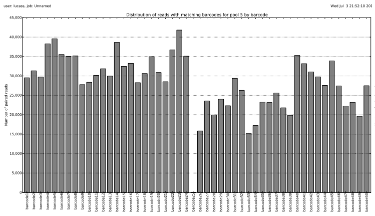

(f) Pool 5

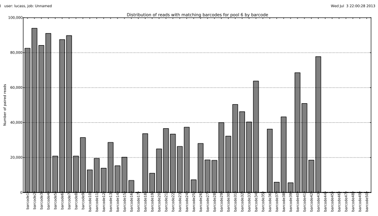

(g) Pool 6

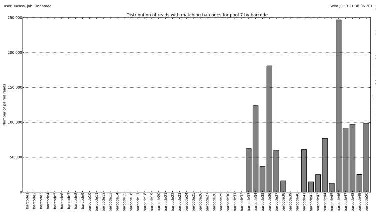

(h) Pool 7

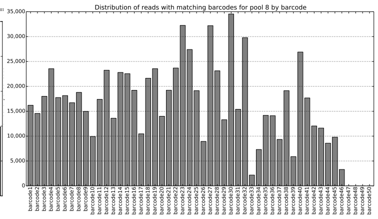

(i) Pool 8
