# Supplementary material for: Microbial Community Composition and Diversity via 16S rRNA Gene Amplicons: Evaluating the Illumina Platform
Source: PLoS One. 2015 Feb 3;10(2):e0116955. doi: 10.1371/journal.pone.0116955 (PMC4315398; doi:10.1371/journal.pone.0116955)
Supplement: S1 Table — The full list of 50 barcodes used along with their nucleotide sequence. (PDF) [file pone.0116955.s013.pdf]

Number	Name	Sequence	Name	Sequence
1	ILL-341F-BA-1	TATCACG	ILL-805R-BA-1	ACTGAGA
2	ILL-341F-BA-2	TACGTCA	ILL-805R-BA-2	ACTGACT
3	ILL-341F-BA-3	ACATCAG	ILL-805R-BA-3	ACTGATC
4	ILL-341F-BA-4	ACACTGT	ILL-805R-BA-4	ACTGTGT
5	ILL-341F-BA-5	ACACACT	ILL-805R-BA-5	ACTCGAT
6	ILL-341F-BA-6	ACACAGA	ILL-805R-BA-6	ACTCAGT
7	ILL-341F-BA-7	ACGATGT	ILL-805R-BA-7	ACTCTGA
8	ILL-341F-BA-8	ACGAGTA	ILL-805R-BA-8	ACTCTCT
9	ILL-341F-BA-9	ACGAGAT	ILL-805R-BA-9	ACTCTAC
10	ILL-341F-BA-10	TACGAGT	ILL-805R-BA-10	TACTGCA
11	ILL-341F-BA-11	ACGCTAT	ILL-805R-BA-11	ACTACGT
12	ILL-341F-BA-12	AGTCTCA	ILL-805R-BA-12	ACTACAC
13	ILL-341F-BA-13	AGTCACT	ILL-805R-BA-13	ACTATGC
14	ILL-341F-BA-14	AGTCAGA	ILL-805R-BA-14	ATGCGAT
15	ILL-341F-BA-15	AGTGAGT	ILL-805R-BA-15	ATGCACT
16	ILL-341F-BA-16	AGATGCT	ILL-805R-BA-16	ATGCTGT
17	ILL-341F-BA-17	TACGCTA	ILL-805R-BA-17	ATGAGCA
18	ILL-341F-BA-18	AGACTCT	ILL-805R-BA-18	ATGAGTC
19	ILL-341F-BA-19	AGACTGA	ILL-805R-BA-19	ATGTGCT
20	ILL-341F-BA-20	AGACACA	ILL-805R-BA-20	TACTGAC
21	ILL-341F-BA-21	AGACGAT	ILL-805R-BA-21	ATGTCGT
22	ILL-341F-BA-22	AGAGTCA	ILL-805R-BA-22	ATCGCAT
23	ILL-341F-BA-23	AGAGATG	ILL-805R-BA-23	ATCGACT
24	ILL-341F-BA-24	AGAGACT	ILL-805R-BA-24	ATCGTGT
25	ILL-341F-BA-25	AGCATGT	ILL-805R-BA-25	ATCAGAC
26	ILL-341F-BA-26	TAGTCAG	ILL-805R-BA-26	ATCACTC
27	ILL-341F-BA-27	AGCAGAT	ILL-805R-BA-27	ATCATGC
28	ILL-341F-BA-28	GTATCAC	ILL-805R-BA-28	ATCTCAC
29	ILL-341F-BA-29	GTCTACT	ILL-805R-BA-29	TACTCGA
30	ILL-341F-BA-30	GTCTCAT	ILL-805R-BA-30	ATAGCGT
31	ILL-341F-BA-31	TAGACTG	ILL-805R-BA-31	ATACGCT
32	ILL-341F-BA-32	GTCATAG	ILL-805R-BA-32	ATACTGC
33	ILL-341F-BA-33	GTCATCT	ILL-805R-BA-33	TGCGATA
34	ILL-341F-BA-34	GTCACTA	ILL-805R-BA-34	TGCTGTA
35	ILL-341F-BA-35	GATACTG	ILL-805R-BA-35	TGCTAGA
36	ILL-341F-BA-36	TAGACGA	ILL-805R-BA-36	TGAGCAT
37	ILL-341F-BA-37	GATGAGT	ILL-805R-BA-37	TGAGTGA
38	ILL-341F-BA-38	GACTATG	ILL-805R-BA-38	TACTCTC
39	ILL-341F-BA-39	GACTCTA	ILL-805R-BA-39	TGAGTAC
40	ILL-341F-BA-40	GACTGAT	ILL-805R-BA-40	TGACACT
41	ILL-341F-BA-41	GACATCA	ILL-805R-BA-41	TGACTCA
42	ILL-341F-BA-42	TAGAGAG	ILL-805R-BA-42	TGTGCTA
43	ILL-341F-BA-43	GAGTCAT	ILL-805R-BA-43	TGTGTCA
44	ILL-341F-BA-44	GAGAGAT	ILL-805R-BA-44	TGTCGTA
45	ILL-341F-BA-45	GCTATCA	ILL-805R-BA-45	TGTCACA
46	ILL-341F-BA-46	GCTACTA	ILL-805R-BA-46	TACTAGC
47	ILL-341F-BA-47	GCTCATA	ILL-805R-BA-47	TCGCATA
48	ILL-341F-BA-48	GCATACT	ILL-805R-BA-48	TCGTAGA
49	ILL-341F-BA-49	GCATCAT	ILL-805R-BA-49	TCAGCTA
50	ILL-341F-BA-50	TAGAGCA	ILL-805R-BA-50	TCAGAGA
